# A Comprehensive Overview of IoT-Based Federated Learning: Focusing on Client Selection Methods

**DOI:** 10.3390/s23167235

**Published:** 2023-08-17

**Authors:** Naghmeh Khajehali, Jun Yan, Yang-Wai Chow, Mahdi Fahmideh

**Affiliations:** 1 School of Computing and Information Technology, University of Wollongong, Wollongong, NSW 2522, Australia; jyan@uow.edu.au (J.Y.); caseyc@uow.edu.au (Y.-W.C.); 2 School of Business, University of Southern Queensland (USQ), Brisbane, QLD 4350, Australia; mahdi.fahmideh@usq.edu.au

**Keywords:** machine learning, federated learning, client selection, participant selection, node selection, device selection

## Abstract

The integration of the Internet of Things (IoT) with machine learning (ML) is revolutionizing how services and applications impact our daily lives. In traditional ML methods, data are collected and processed centrally. However, modern IoT networks face challenges in implementing this approach due to their vast amount of data and privacy concerns. To overcome these issues, federated learning (FL) has emerged as a solution. FL allows ML methods to achieve collaborative training by transferring model parameters instead of client data. One of the significant challenges of federated learning is that IoT devices as clients usually have different computation and communication capacities in a dynamic environment. At the same time, their network availability is unstable, and their data quality varies. To achieve high-quality federated learning and handle these challenges, designing the proper client selection process and methods are essential, which involves selecting suitable clients from the candidates. This study presents a comprehensive systematic literature review (SLR) that focuses on the challenges of client selection (CS) in the context of federated learning (FL). The objective of this SLR is to facilitate future research and development of CS methods in FL. Additionally, a detailed and in-depth overview of the CS process is provided, encompassing its abstract implementation and essential characteristics. This comprehensive presentation enables the application of CS in diverse domains. Furthermore, various CS methods are thoroughly categorized and explained based on their key characteristics and their ability to address specific challenges. This categorization offers valuable insights into the current state of the literature while also providing a roadmap for prospective investigations in this area of research.

## 1. Introduction

IoT refers to a network of interconnected devices, sensors, and objects that collect and exchange data. These devices can be anything from smartphones and wearables to smart home appliances, industrial sensors, or autonomous vehicles. The convergence of the IoT and ML presents a compelling alliance with the capability to revolutionize IoT applications across diverse sectors. IoT devices continuously collect extensive data from various sources such as sensors and cameras. ML algorithms can effectively utilize this data to derive valuable insights, enable real-time decision-making, and enhance process optimization. In ML used in conjunction with the IoT, there is a concern about the amount of data involved in the training process, especially when the data are sensitive [1,2,3]. One of the most promising solutions to the isolated data island [1] problem is FL, where many clients ranging from edge devices to IoT devices collaboratively train a model under the orchestration of a central server. In FL, local data do not need to leave the clients. This means that ML training can be performed without transferring client data from their original location to the servers [4]. Using FL, clients can create centralized, robust, and precise local models that are sent to the server [5]. In this way, it reduces privacy concerns while allowing users to keep their information private [6,7]. In addition, it speeds up and improves local model training [8]. For instance, Dayan et al. [9] performed a representative study of an extensive, real-world healthcare FL examination across several sites and datasets. In their study, FL provided fast cooperation and improved results with no data transferred between the participating clients. They concluded that rapid and collaborative development of AI methods in healthcare can be accomplished with FL [9]. FL is also useful in the following areas:

- **RS:** RS are information filtering systems designed to anticipate user preferences and offer personalized recommendations. Employing FL in RS yields numerous benefits and enhancements, including the delivery of efficient and privacy-preserving personalized recommendations to users across diverse platforms and devices [10].
- **IoT applications:**
  ➢ **IoV:** FL also has the potential to bring about a revolutionary transformation in the automotive industry and the development of intelligent transportation systems [11].
  ➢ **MEC:** The incorporation of FL into MEC is anticipated to have a crucial impact in achieving efficient and privacy-preserving intelligent applications at the network’s edge [12].
  ➢ **IIoT:** The integration of IIoT with FL has the potential of revolutionizing industries and streamlining industrial operations. IIoT involves a network of interconnected devices, sensors, and equipment in industrial environments, enabling data collection and exchange. On the other hand, FL is a privacy-preserving machine learning approach that facilitates model training across distributed devices without the need to share sensitive raw data [13].
  ➢ **IoHT:** The integration of IoHT with FL is a solution to enhance healthcare practices, enabling advancements in remote patient monitoring, disease prediction, and treatment optimization [14].

Despite the advantages of FL, there are some serious challenges such as expensive and inefficient communication [15,16], statistical heterogeneity, poor data quality [17], privacy concerns [18,19,20], and client heterogeneity [21]. To solve these challenges, numerous investigations and studies have been performed. For instance, the authors in [22] focused on the communication efficiency and client heterogeneity problem of FL and proposed a new solution. However, the proposed solution suffers from a growing number of clients. The issue was solved by increasing the computation capability of clients [23]; however, this solution increased costs. In relation to the problem of poor data quality, an intelligent medical system was studied [24]. In such systems, different types of diseases have different data structures and non-IID data, so training heterogeneous datasets is a major issue [17,25]. To address this challenge, a solution of enabling local model training and only communicating model updates is proposed [26,27]. Researchers also have proposed various training methods, such as clustering of training data [28], multi-stage training and fine-tuning of models [29], and edge computing [30]. However, these approaches are still immature, and dealing with data quality while preserving model performance remains an open problem [12,31]. While FL does not require raw data to leave client devices, it is still possible for the information to leak from local model gradient updates [28,32]. In addition, the existence of malicious clients in the training process can reduce system reliability and poison model performance. This can happen by disrupting the training process or providing false updates to the central server [6]. Hence, there is a need to develop and employ more comprehensive and robust solutions for enabling FL to better handle its challenges.

In recent years, client selection (CS) methods have been introduced as one of the essential solutions to alleviate the above challenges [33,34,35]. Overall, the server evaluates a client’s performance based on information from the local models it receives [36]. Due to bandwidth limitations [37] and the availability of many clients [34], a selected subset of them can take part in the process at each training round [37]. It should be noted that clients usually have significant differences in terms of resource constraints, heterogeneous hardware conditions, and data resources in many procedures [38]. The CS process actively selects participating clients based on predefined criteria for each training round. Researchers have demonstrated that different criteria can be adopted to achieve objectives like fast convergence, low communication costs, optimal final model, and maximizing the overall performance of a model [1,34,35]. For example, the authors in [1] show that compared with the vanilla CS algorithm for FL, given the same number of rounds, their proposed CS solution (RBCS-F) decreased the training time. However, it is not clear why and how CS can enhance global model accuracy, and how to ensure a secure, reliable, and fast CS method that can cope with non-IID, unbalanced data, and bandwidth restrictions. More specifically, unlike data centers with unlimited resources and adequate bandwidth, clients in FL have resource constraints and are heterogeneous hardware systems, which can lead to training latency and significantly decreases the FL performance [35,39]. For instance, sensor faults and environmental restrictions can cause a cluster of mislabeled and non-IID on mobile clients, resulting in reduced local learning qualities [17,25]. Every client has differences in its dataset distribution, performance, model parameters, available computing resources, energy consumption, etc. As a result, they have different impacts on the round of training, the convergence speed of the FL process, and the global model’s accuracy. Furthermore, bandwidth limitations [5,40] pose a risk in the training of all clients and uploading all parameters to the server [41]. From a model owner’s standpoint, it is important to know whether CS has a significant influence on reducing the training time of a model. It is important to know whether CS can enhance the convergence rate, achieve more stable training, and improve final accuracy. In relation to these considerations, CS can be an effective solution for FL optimization [34,41]. Under large-scale FL scenarios, finding a suitable CS technique requires a massive search area with a non-polynomial time complexity that cannot be performed in real-time. As a result, to achieve a high standard of FL, the CS process and its categories are essential to selecting the best clients from the pool of candidates [28]. Therefore, it is necessary to thoroughly review, analyze and categorize this research domain. So far, different aspects of FL development have been examined and reviewed in the literature. However, to date, there is limited work on systematically reviewing the CS process and existing CS methods, along with their potential challenges, characteristics, and shortcomings. This paper provides an in-depth overview and detailed analysis of CS categories based on existing research. The aim of this is to assist industry practitioners and researchers in exploring the challenges and potential gaps related to CS methods and their development. The main contributions of this systematic literature review (SLR) are as follows:

➢ A thorough SLR is presented that examines the challenges of FL in adopting CS methods that can be used to aid future research and development of CS in FL.
➢ A detailed overview of the CS process, including its abstract implementation and characteristics, is presented that can be used in various domains.
➢ Different CS methods are categorized and explained based on their main characteristics and the challenges they solve. This provides insight into current literature and provides a plan for future investigations on this topic.

This article is organized as follows. Section 2 presents a comprehensive background including the definition, challenges, and importance of CS in FL. Then, Section 3 discusses the research methodology. In Section 4, the challenges associated with FL, an overall structure-based review of CS as a potential solution to these challenges, and the prominent factors impacting a model’s performance are discussed. This is followed by Section 5, which presents different methods for enhancing the performance of FL based on CS. Additionally, major side effects and categories of CS methods are explained and analyzed. Finally, Section 6 future trends and directions, and Section 7 concludes the paper’s outlines. In general, this work presents a comprehensive study of the overall vision, structure, configurations, and significant structures associated with CS for FL.

## 2. Background

### 2.1. Client Selection

The increasing number and type of network services and the proliferation of mobile edge have prompted the deployment of IoT [2] devices with advanced sensors, computing, and communication capabilities for crowd-sensing tasks [42]. The advent of AI has led to significant developments in numerous modern applications, such as air quality, weather monitoring, and video surveillance [1,2]. Nowadays, ML algorithms and intelligent applications have made it possible to analyze various types of data, including text, numeric, photographs, videos, and locations, from different IoT devices [20,43,44]. However, ML typically employs centralized data, which raises several problems [45]. Data privacy [46] is a major problem since data cannot be transferred from the devices. There are also challenges related to massive scale [47] and optimization [48]. In addition, non-uniform data distribution refers to a significant discrepancy between the size and distribution of data (texts, images, and videos) stored on devices, which makes data transfer challenging [3]. This is compounded by the limited bandwidth between devices and the server. To overcome these ML problems, FL was proposed by Google [8]. FL means that multiple entities are able to create common ML without data sharing. This addresses critical issues such as data privacy, access rights, and access to non-uniform data distribution data on a massive scale. FL can be classified into two types based on the participation of clients and the training scale:

➢ Cross-device FL with millions of clients such as smartphones, wearables, and edge device nodes, where each client typically stores local data [49].
➢ Cross-silo FL in which the client is typically a company or organization, with a small number of participants and a huge amount of data, and each client is expected to participate in the entire training process [18].

Using cross-device FL, the parties, entities, or clients can share trained and updated models more easily since the bandwidth obstacle in ML is removed [41]. In FL, raw data from the clients do not need to be transferred to the central server to achieve an aggregated final model because all training is conducted locally on the clients [6,7]. To be specific, only the post-trained model or parameters are sent to the server once the training process has been completed by the local client nodes, which in turn protects the privacy of the data owners [33]. Then, the model parameters or the post-trained model in FL should be optimized with minimal loss by using a gradient approach, such as stochastic gradient descent (SGD) [45]. In basic FL, randomly selecting clients from a list of candidate clients is not the best method to achieve an optimal global model [7,8,33]. Local clients train the global model by using local data. This step is conducted by utilizing aggregated model updates before committing the model updates to the server for aggregating the final model. The global model is then adapted before being returned to each device for the subsequent iteration [8]. So, the convergence speed of the model can be affected by the number of participant clients, training iteration, resource allocation, data diversity, and aggregation method. In this process, hardware issues and data resources can significantly impact learning performance. In other words, end client nodes usually have different computation and communication capacities and are connected in an unstable environment. There is a risk of stragglers, which means that some clients with low-level resources are unable to complete their training within the deadline. Moreover, mislabeled and non-IID data [3,25] with different data quality are frequently gathered from clients due to sensor flaws and environmental restrictions, leading to various local learning shortcomings. To deal with these challenges, it is necessary to employ an efficient method to select appropriate clients during FL training. Therefore, greater understanding and research on the CS process are needed to optimize FL effectiveness and acceptable accuracy [8], leading to increased overall performance. For this, a comprehensive review of the CS process, methods, and categories will provide much-needed insight for the research community. So far, several review papers have been published on this topic, presenting proposals, methods, and practical examinations [6,50,51]. However, a rigorous and well-defined SLR is required to classify and analyze the most important and latest research papers on this topic. Hence, an SLR of CS concepts, developments, and methods based on qualitative analysis from a design perspective is presented in this article for the first time. This review paper addresses the following RQs:

(1) **RQ1:** Why and how can adopting an appropriate CS method optimize the overall performance of FL?
  (a) **RQ1.1:** In which aspects should FL be improved?
  (b) **RQ1.2:** How can CS help resolve the FL challenges?

(2) **RQ2:** From the structural perspective, what are the main pros and cons of different CS methods?
  (a) **RQ2.1:** How can different methods be categorized in different terms?
  (b) **RQ2.2:** What challenges have been addressed with CS methods?

By addressing the above-mentioned questions, this paper provides insights into current research gaps and future research directions.

### 2.2. Related Surveys

This subsection aims to summarize and discuss the most relevant survey work related to the RQs. As mentioned in Section 2.1, from the aspects of scale and training, there are two main types of FL, namely, cross-silo FL and cross-device FL. Cross-silo FL aims to foster collaboration among several organizations at a large scale, while cross-device FL focuses on ML across large populations, such as mobile devices [3,18,49]. This paper mostly focuses on cross-device FL; thus, this type of FL and its related publications are discussed. In different application domains of cross-device FL, such as IoT devices, mobile edge computing, and cloud computing, there are severe challenges like highly heterogeneous data, heterogeneous client configurations, privacy, and communication efficiency issues [18,38,52] among clients (all mobile or IoT devices). Mishandling these challenges can adversely affect the performance of FL. Hence, CS methods are used to help solve these challenges [6,34]. Employing an effective FL CS method, handling the heterogeneity of data and clients, reducing training overheads, guaranteeing privacy, efficient communication, strengthening robustness, and improving model accuracy can be achieved. Thus, the development of FL based on improved or new CS methods is increasingly being studied within the research community [18,51,52].

In Table 1, different review papers are compared based on their main features and criteria. As listed in Table 1, there are three kinds of review papers written on this topic:

➢ Focusing on FL challenges without considering different CS methods: Li et al. [19], Zhang et al. [24], Liu et al. [27], Wen et al. [32], Zhang et al. [36], Nguyen et al. [53], Antunes et al. [54], Campos et al. [55], and Banabilah et al. [38] focus on FL challenges from the perspectives of IoT devices, IoT, privacy applications, 6G communication, privacy protection, intelligent healthcare, healthcare applications, intrusion detection in IoT, and edge computing respectively.
➢ Reviewing FL challenges and introducing CS as a solution without discussing its challenges: Lo et al. [18] examined the development and challenges of FL systems from the software engineering perspective.
➢ Focusing on the challenges of CS methods: Only two papers focus on CS and its importance for FL. In [41], only system and statistical homogeneity challenges are discussed without considering fairness, robustness, and privacy issues. In contrast, the authors in [6] briefly examines the critical challenges of CS methods extracted from current research, compares them to find the root causes of the challenges, and guides future research. However, it is not a comprehensive survey and does not contain data privacy issues or the design architecture of CS methods.

Consequently, it is important to present a comprehensive and organized review that covers all of the criteria listed in Table 1. As stated, the literature on CS is relatively recent and has been advancing rapidly. Also, there is no thorough understanding of FL challenges and CS as a solution from the structural design lens to respond to these challenges. Moreover, the role of CS methods in improving convergence speed, model performance, decreasing communication costs, and attaining an optimal model has not been clearly understood. For addressing these research gaps, this review paper supplies an in-depth understanding of the CS process from the design perspective along with specifying the importance of CS methods as an effective solution for FL challenges. To be more specific, it demonstrates the significance of CS on the accuracy of an FL model through various techniques, including characteristics associated with each technique. This paper systematically demonstrates how CS can solve FL challenges, how it is evolving, and its challenges and opportunities. The aim of this is to assist practitioners in selecting the most appropriate CS method for their applications and to encourage investigators and researchers to gain a deeper understanding of this exciting research topic. This will undoubtedly shed light on the existing research gaps and future research directions.

## 3. Research Methodology

An SLR is a comprehensive scientific method of investigating, determining, and evaluating research questions. It aims to determine, diagnose, and evaluate research responses corresponding to the specified RQ containing high-quality findings. Other than providing a thorough review of relevant studies, an SLR also determines current study gaps, supplies a basis for additional investigations, and elucidates new phenomena [56].

**Figure 1:**
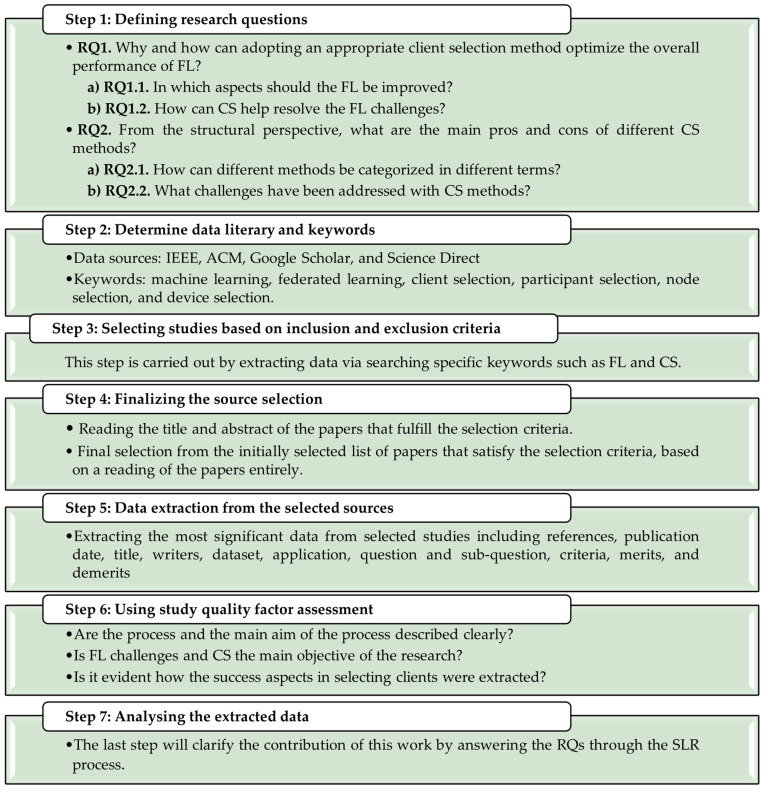
Conceptual Framework.

In this paper, after collating research papers through manual and automated searches using the SLR research methodology, the latest and most important literature on CS is categorized and analyzed. Figure 1 summarizes the steps and methodology used in this study to produce this comprehensive SLR. The following subsections explain these steps in more detail.

A. Defining research questions.

As the first step of a research methodology, it is required to define the RQs. In Figure 1, the RQs of this study are listed.

B. Determine data literacy and keywords.

To answer the research questions, it is essential to choose the best and most helpful and valid data sources [56,57]. Here, the needed data were gathered from solid and well-known databases like IEEE, ACM, Springer, and Elsevier. Based on the RQs, a set of search queries, related abbreviations, and alternative synonyms such as “machine learning”, “federated learning”, “client selection”, “participant selection”, and “node selection” were used for gathering data from those databases. Conducting a keyword search yielded an initial pool of 130 resources. This study aims to encompass a comprehensive overview by incorporating scholarly publications from esteemed journals and reputable conference proceedings, ensuring the inclusion of high-quality academic work.

C. Selecting studies based on inclusion and exclusion criteria.

The inclusion criteria in this paper contain which type of research literature, from papers to technical reports, can be utilized for extracting data by searching specific terms [56]. For instance:

- The papers explicitly addressed challenges in FL related to CS.
- The papers were published in internationally recognized computer science journals and conferences. These publishers contribute to computer Science applications, and algorithms are used to structure the logic of their programs, perform computations, manipulate data, and control the flow of execution to simplify the CS process.
- The papers were written in English.

Moreover, the studies irrelevant to the scope of this paper were excluded and are based on the following categories:

- Papers without evaluation results, such as white papers or short papers.
- Papers that provided background information on FL.
- Papers without peer review, such as theses.
- Papers not written in English.

By applying rigorous inclusion and exclusion criteria, the number of resources was narrowed down to 86.

D. Finalizing the source selection.

First, the primary source selection was performed by reading the title and abstract of the papers. Then, the final selection from the shortlisted papers is made based on details of their content and contributions. A meticulous examination of the title and abstract of the remaining papers in accordance with the selection criteria resulted in a total of 80 papers. Finally, after an in-depth evaluation of the papers in the initially selected list, 69 papers emerged as the final selection that met all of the selection criteria.

E. Data extraction from the selected sources

In this step, the critical information of each paper was extracted and gathered, which contains their references, publication date, title, authors, datasets, applications, questions and sub-questions, criteria, merits, and demerits.

F. Using study quality factor assessment.

To assess the selected papers, three main quality factors were used, which are listed in Figure 1. This assessment guarantees that the steps taken up to now, i.e., steps 1–5, have been carried out correctly.

G. Analyzing the extracted data.

Table 2 categorizes the selected papers based on the RQs defined in this paper. In this table, it is clear where each paper falls within the RQs. As can clearly be seen, this review is a novel attempt to contribute significantly to the understanding of CS. Clearly, this survey outweighs the previously published studies in terms of scope, depth, and coverage, since it aims to answer all of the defined RQs at the same time.

## 4. Discussing CS’s Impact on FL Challenges and Its Challenges

### 4.1. FL Structure and Its Challenges

In this part, the overall structure of FL along with the main challenges of FL are presented.

**Figure 2:**
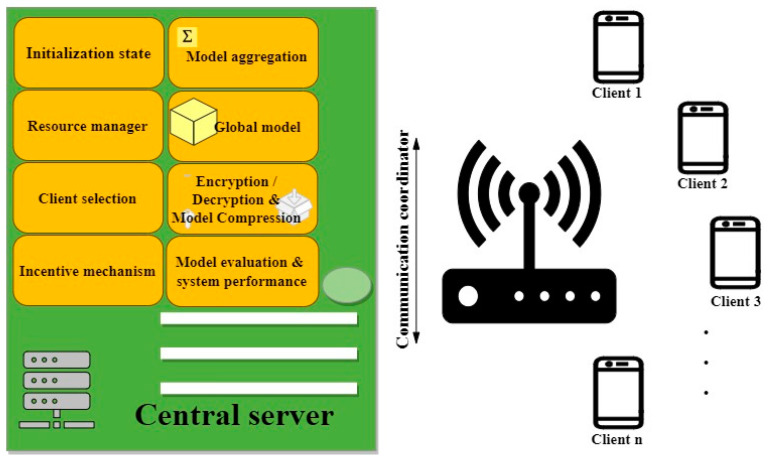
The general flowchart of the FL including server initialization, resource management, client selection, incentive mechanism, model aggregation, global model, encryption/decryption, model compression, and model and system evaluation.

**Figure 3:**
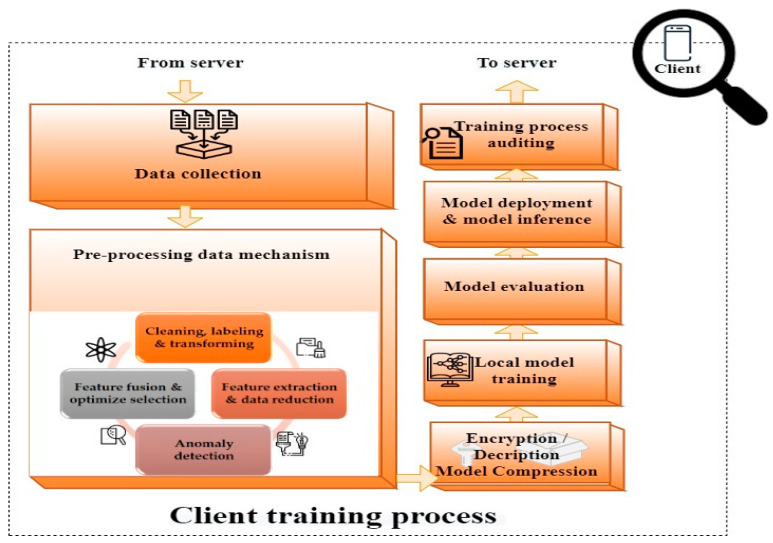
Client training includes data collection, pre-processing data, encryption/decryption compression, model training, model evaluation, model deployment, and audit training process.

As mentioned in Section 2, FL was developed due to the challenges of ML, including the lack of privacy in transferring data, its massive scale and heterogeneity, and non-uniform data distribution. The general flowchart of FL is shown in Figure 2 and Figure 3. The server (Figure 2) and the client (Figure 3) are two significant parts of FL. These two significant parts are explained in the following.

- Central Server. The server is one of the key parts of FL. The server initializes the process by completing a foremost global model using a sample dataset generated by itself or by collecting data from clients [62]. In some FL systems like in [75], clients start the global model. Then, an encrypted and compressed global model is broadcasted to clients based on an examination of the available clients [50,51,63] or based on the participating clients’ performance in the last step [64]. After that, a trained local model can be collected from all clients or only the participating clients accordingly. The communication coordinator is an administrator that provides a channel between the server and multiple clients for communication [37]. It is also possible to collect local models either synchronously or asynchronously [57,66]. In contrast to synchronous, an asynchronous scheme means that clients do not need to wait for each other to synchronize. When the server receives all or part of the updates, it performs model aggregation. After that, clients are notified of the updated global model. In the end, the evaluation part assesses the system performance of the process. This process continues until convergence is reached. In addition to orchestrating the exchange of model parameters, FL also has other parts, especially a resource manager and a CS process [18]. The resource manager is to make the best use of resources. It is the administration system for the optimization of resource consumption and control of the allocated resources of clients. The result of this is reflected in the CS mechanism for selecting suitable clients to conduct model training and reaching desirable system performance [68]. In addition, clients may be motivated to participate through incentive mechanisms [71,72,73].
- Clients. As another important part of FL, clients train local models at each iteration using their local data. To begin (see Figure 3), each client gathers and pre-processes its data through various steps, including cleaning, labeling, data augmentation, data transformation, feature extraction, data reduction, anomaly detection, feature fusion, and selection optimization [20]. Then, each client receives the global model and initiates the operations of decryption, decompression, and parameter extraction from the global model. This step is followed by performing local model training by clients. After being trained for multiple rounds [77], the model is evaluated by the client and audited as being complete. Model evaluation is to ensure that the model has reached the expected level of performance. This step is followed by model deployment and model inference. After this step, the model is compressed to acquire a sufficient level of performance and to decrease communication costs [63,72,74]. Encryption is applied to the local model before it is uploaded to secure the process and the data. Then, the local models are sent to the server to aggregate the results [78].

It is clear that FL has a comprehensive and coherent structure. Other than its advantages, FL also suffers from severe problems, which are briefly explained as follows:

#### 4.1.1. Expensive and Inefficient Communication

Communication is a fundamental problem in federated networks. Due to communication costs and privacy concerns in federated networks, data generated by each client node must remain local [6]. Instead of forwarding the complete dataset through the federated network for model fitting, clients transfer information or model updates repeatedly to the server during training. This means that several rounds of training are needed before the system converges to achieve the required level of accuracy. Hence, the federated network may be overloaded because of numerous clients sending their updates to the server. Moreover, network communication speed cannot be guaranteed because a federated network may contain many smartphone clients, which have limited communication bandwidth, energy, and power, and there are different transmission standards such as 3G, 4G, 5G, and Wi-Fi. As a primary solution, expensive communication can be employed to avoid overload and achieve high data transfer speed simultaneously. However, this is not desirable. As an alternative, a desirable solution is for a more efficient communication method to be developed and used. Hence, the design of a method with high communication efficiency is essential for practical FL [38]. So far, some suggestions to achieve this aim have been presented, including local updating techniques, compaction strategies, and decentralized training [6,28]. However, these solutions still have efficiency problems in terms of communication, and there is large room for further research.

#### 4.1.2. Statistical Heterogeneity

Statistical heterogeneity is the second challenge in FL. It refers to the distribution of data volume and class distribution variance among clients. It contains two factors: data quality and non-IID heterogeneity [18]. Variations in data quality can arise from diverse data samples used during training for each client in each iteration round [8,41]. Furthermore, each client owns a small portion of data, which it independently uses for training [58], so differences in unbalanced data classes (model parameters) result in fluctuated distribution reflecting non-uniform distribution [25] and local data overfitting, which are two issues that lead to non-IID. Model training latency and accuracy can be affected by these factors [6,34]. As a result, it is important when each client trains on local data independently to create a local model, and these models must be very flexible to reduce the statistical heterogeneity risk. Some methods have been suggested to control this, such as data modeling for heterogeneous datasets and a converged dataset for non-IID [53]. However, it is possible to design better solutions to balance accuracy and data heterogeneity efficiently.

#### 4.1.3. Client Heterogeneity

Differences in the client resources, such as computation, storage capabilities, and battery level, mean heterogeneity of clients, which is the third challenge in FL. These differences are due to various reasons. First, there may be differences in hardware, which affects the capacity of CPUs and memory that run AI models. Training models may take a long time since AI instances cannot fit into the memory of AI accelerators, or it is possible that AI model operators are not supported on devices [24,55]. Battery power can be the second cause of differences among clients. The battery power level of clients depletes when running applications and taking part in the training process [59,60].

Due to the above-mentioned causes and network status [19], only a fraction of clients can be active simultaneously. Ignoring client resource capabilities affects dropouts of the model during the training process, leading to training deficiency, which impacts the accuracy of the model. So, FL should cater to the following considerations to reduce the risk of client heterogeneity:

- Expect an inferior portion of the participation.
- There is a need to consider this attribute specifically.
- Tolerate faults in heterogeneous hardware. It is a vital attribute of classical distributed systems to support fault tolerance, including Byzantine formalism failures [88]. Since some remote clients may drop out before completing training, fault tolerance becomes even more critical. For instance, suppose the failed clients have specific data properties. Ignoring such client failures, like in FedAvg [18], may lead to bias. FedAvg is difficult to analyze theoretically in such realistic scenarios and thus lacks convergence guarantees to characterize its behavior.
- Be sufficiently solid to drop clients in the transmission. As there is a risk of dropping clients during FL due to computational capability or poor network connection, the FL process should be solid enough even when encountering this issue [59].
- Asynchronous communication. Due to client variability, they are also more exposed to stragglers [57]. Stragglers mean that some clients with low-level resources are unable to complete their training within the deadline. The use of this scheme, particularly in shared memory systems, is an attractive technique to mitigate stragglers [19,59], although they generally use boundary-delay assumptions to deal with staleness. Li et al. [39] also proposed a FedProx optimization method in FL to cope with heterogeneity, but it lacks formalization. Although asynchronous FL has been demonstrated to be more practical even with its restrictions [59], new solutions to ensure more expected performance are under-explored.
- Active device sampling. Each round of training in federated networks typically involves just a small number of clients. Nevertheless, most of these clients are passive in that round and each round does not aim to control which clients participate.

As a result, as was explained, some techniques have been examined in recent studies. However, providing the mentioned attributes in a complete solution is of high importance.

#### 4.1.4. Data Privacy

An FL training process should keep user details private since FL aims to solve data privacy issues in ML [19,55]. As previously stated, FL is a step toward preserving the privacy of data generated on clients while transferring model changes instead of the raw data. Nevertheless, this communication may still disclose data and bring privacy risks. There are two privacy strategies in the FL structure, global privacy and local privacy [18,19]. Current strategies improve FL privacy by utilizing secure multiparty computation clients or differential privacy that preserve privacy at the client level rather than after data aggregation. These techniques mainly decrease the performance of the model or the efficiency of the design. In FL, the server may fail to aggregate the global model when clients upload untrustworthy and unreliable data. It is, therefore, crucial to find trustworthy and reliable clients in this scheme. A reputation measure was proposed in [28] to identify highly reliable clients and calculate their trustworthiness rating during the model update process.

In summary, this section corresponds to RQ1.1 and explains the significant challenges of FL in research. Theoretically and empirically, understanding and addressing these challenges are significant difficulties in FL approaches.

### 4.2. CS in FL

**Figure 4:**
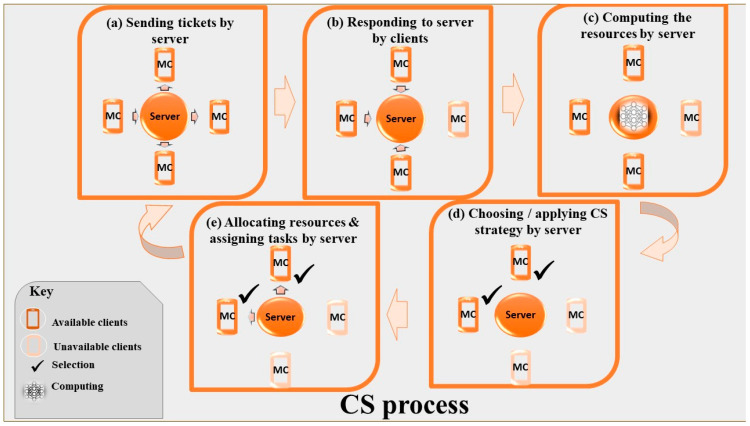
CS process: (**a**) The server sends a ticket to the clients for detecting and monitoring clients. (**b**)The available clients for model training respond. (**c**) The server computes the available resources. (**d**) The server selects a strategy to opt for participation. (**e**) Resource allocation is conducted, and tasks are assigned to participants.

As seen in Figure 2, the server initiates model training and orchestrates training rounds while clients carry out local model training. By choosing an appropriate CS method, suitable clients can be selected for evaluating the model and system performance [68]. Furthermore, Figure 4 shows the detailed CS process in a simple and categorized way. This provides a better and more general understanding of the CS process and its different parts and steps. The server sends a ticket to the clients for detecting and monitoring clients. The online clients who want to take part in the model training process respond to the server’s request. Then, the server computes the available resources and uses a specific strategy to choose participating clients. Resource allocation to the selected clients is conducted for the training process. After that, tasks are assigned to participants in two ways. First, use a hybrid algorithm and scheduling to repeat the chosen CS method until convergence is achieved. Second, the process is repeated for each iteration round. Scheduling the task can improve the system’s efficiency [26,85]. However, the implementation of this strategy is sometimes impossible, especially in a volatile environment. According to the explanations in this part, the mentioned challenges in the FL are in need of a solid solution. Focusing on the CS process can be a suitable approach to address the mentioned challenges in FL. As it is evident, RQ1.2 was addressed in this section.

## 5. Pros and Cons of Different CS Methods

### 5.1. CS Methods

The main aim of this part is to describe different CS methods.

(1) Client selection methods based on the probability of selection: four CS methods based on the probability of selection at each round are presented as follows.

**Random selection**

There are several CS methods in FL, but randomly selecting clients is the conventional approach [86,87,88]. Based on the FedAvg method [18], all clients will have the same probability of being selected for model training. In this method, aggregation is inefficient as this method ignores value differences among clients. In this method, each client trains its local model using its own data and then sends the updated model to the central server for aggregation. During aggregation, the central server simply averages the model updates from all clients and uses this average to update the global model. The inefficiency arises because FedAvg treats all client updates equally, regardless of the amount or quality of data each client has. Some clients might have more diverse or informative data, while others might have noisy or less relevant data. By blindly averaging all updates, valuable information from high-quality clients may be diluted or lost in the process. Additionally, this method does not consider the data heterogeneity of clients. The weak point of this method in a distributed computing environment is its high communication costs because the central server receives updates from distributed clients on a fixed bandwidth [5]. It is possible to save transportation costs by randomly selecting a part of the updated model parameters for transportation by random masking [33]. However, it has restrictions, which we will discuss in more detail in the evaluation section.

**Greedy selection**

This method chooses clients with high-level quality grades and low expenses. It utilizes a heuristic method to characterize the quality rate of each client [33,85]. Each client employs a tiny subset of local data to train the global model and evaluate the FL platform model. Recently, this method has been widely used to evaluate the quality of budgeted incentive mechanisms in selecting the most influential clients for incentives [62]. In other words, this method selects the set of clients with the most considerable collaborative feedback. The FedCS algorithm proposed by Nisho [8] is mainly based on the greedy method. This algorithm is a typical example that is adjusted by picking the clients with the most significant average contribution instead of selecting the clients that complete the training in less time. This approach of CS prefers clients with high-level efficiency during each iteration training round. Then, it can effectively enhance the aggregation efficiency of FL models by completing the training model quickly and before the deadline. In this method, data collection is performed in FL regardless of existing clients in a federated network. As the amount of data varies significantly in different clients in FL, the data are non-IID in real-world datasets. Similar to the random method, the quality of client data is neglected [8,18]. Accordingly, they cannot reduce the number of clients selected with low-quality data, resulting in low-level accuracy for the global model and gradual convergence. Choosing superior clients accelerates global model convergence and improves global model accuracy along with keeping bandwidth boundaries. This is the primary objective of FL CS.

**Clustering selection**

In this method, clients that train the model are clustered according to their attribute similarities, including their resources, allocated data, characteristics, location, segment similarities, and gradient loss, to enhance the overall model efficiency and boost model training performance. In other words, k-center grouping is performed on the set of clients before training, and then the closest clients to the center client of each cluster are assigned to the cluster and the model training is conducted based on the clusters [13,89,90].

**Multi-Armed Bandit (MAB)**

MAB is mainly used to get the root of repeated discovery situations in which a player (in the FL scenario, typically represented as the server) encounters a situation where it must choose from multiple arms (corresponding to the clients). The player honors the related reward (refer to model performance in FL) when an action is taken (choosing specific clients to participate in model training at each iteration round). Boosting the total prize and making sequential decisions simultaneously is the MAB’s primary goal. Players should examine the surroundings to gain more knowledge on each training round, recognize activities that boost the chance of achieving higher rewards, or exploit existing knowledge to execute the actions that reasonably worked in the past. This method has been used to design client scheduling [1,7,59] or in the CS process [91,92,93]. Three main categories arise from the proposed procedures to decrease the training latency in FL:

1. Update compression (quantizing gradient is a solution for efficient communication).
2. Over-the-air analysis [63].
3. Reducing transmission (periodic updates of model parameters to lessen the transmission expenses) [34,59].

To summarize, CS method categories are described in this section in response to RQ2.1. These methods have some merits and demerits that we will discuss in the evaluation section.

### 5.2. CS Side Effects

This section describes the side effects of CS methods. Clearly, the implementation of CS methods improves the overall performance of FL in terms of client heterogeneity, statistical heterogeneity, and data quality. However, it is noteworthy that employing these methods may cause or intensify some side effects in FL. A brief explanation of these problems is as follows.

- **Fairness:** Fairness means that every client has an equal chance to be selected for training. When fairness is ignored, the server may prioritize the client with a different dataset size but in a shorter response time. This may significantly affect the training performance. So, clients with insufficient abilities have a lower chance of being selected to participate in the training process, which may lead to bias and low-level model accuracy [1,41]. Fairness boosts the accuracy and speed of convergence of models by enabling clients with various datasets to participate in the FL [34,35,59]. Consequently, all end devices should be involved in the FL process to decrease model bias.
- **Trustworthiness:** Because the FL server is unaware of the local training procedure, malicious clients can launch attacks and manipulate the training outputs. A primary priority should be recognizing and removing malicious clients from the procedure [6].
- **Dynamic environment:** This means that because of the existence of deficiencies, including high mobility, poor network conditions, and energy constraints, some clients might not be available to take part in model training [35,49,59]. Moreover, channel fading in wireless networks may result in losing some local model updates. Therefore, a dynamic condition with high-mobility devices and volatility including client population, client data, training status, and biased data [84] significantly impacts the performance of the CS process and FL.

This section clarifies the most significant side effects in CS such as client heterogeneity, statistical heterogeneity, data quality, fairness, trustworthiness, and dynamic environment (addresses RQ.2.2).

In Table 3, all of the findings and results of CS categories, along with each side effect, main characteristics, application, strategy of each source, and evaluation metrics of each work, are presented. It should be noted that the evaluation metrics are discussed in more detail in Appendix A.

### 5.3. Overall Evaluation of Different CS Methods

- **Clustering methods**

In typical dynamic FL training and clustering methods, FL clients display system and statistical heterogeneity. The main issue in data heterogeneity in clustering is non-IID data issues [10,13,28]. The clustering method can be based on training data [31,89,90] or based on the location of clients and the required skills and efficient collaboration among each other [2,13]. Some work performed clustering if necessary [28] and handled varying client populations. This provides distribution imbalance while its extent in conjunction with privacy strategies and compaction mechanisms is unclear. One work used the successive non-convex penalty (SNCP) approach as a performance evaluator, which can reduce communication costs [90]. However, it cannot handle outlier and noisy data. Some works [28,90] use multi-task learning in times when the clustering structure is ambiguous [31], but it goes with high communication and computation overheads. This challenge has been resolved in [10], and the mentioned method is only suitable for use in the risk functions context and evaluates the similarity of the loss value as a technique of secure data similarity evaluation. The authors in [13] address the divergence issues in class distributions by using a gradient-based binary permutation algorithm (GBP- CS) and tackle the issue of robust FL in a heterogeneous setting by having a functional convergence rate compared to FedAvg. Such methods are time-efficient models along with high-level efficiency.

- **Greedy methods**

In greedy or dynamic methods, resource constraint issues [87,88] contain bandwidth allocation issues [5,40], communication cost issues [33,85], limited computational resources issues [8,42,85], and the energy consumption of selected clients [26,42], which can lead to low accuracy and high convergence time and latency. The authors in [40] proposed a novel perspective to resource allocation in WFLNs, realizing that learning rounds are temporally interdependent and have varying significance toward the final learning outcome. It is adaptive to varying network conditions, and it can enhance the training loss and model accuracy and reduce energy consumption. However, in this method, participation rounds of clients are limited because of the limited battery energy of clients. Clients in a wireless network are limited by finite wireless bandwidth in each iteration, with an adaptive choice to unstable phases of wireless channels. Although they reasoned that always picking the highest number of clients is not necessary, some other work [8,40] considers maximizing the number of the selected clients in each round to upload their local models before the deadline. In another paper, the authors offered a novel strategy [26] to choose fewer clients in earlier global iterations and more clients in later global iterations in the same period of training time. This can increase model accuracy and reduce training loss when compared to choosing more clients at first. Because it overlooks the local data quality of clients and cannot decrease the number of client selections with low-quality data, the global model needs to be more accurate, and convergence needs to be faster. Neither CS nor resource management solutions were discussed in terms of how they affect the convergence and accuracy of global models. Likewise, [87] ignores client data quality, so it is unable to decline client selections with low-level data quality and does not consider the clients’ waiting time leading to clients’ latency. However, it considers client channel conditions and the importance of their local model updates. The authors studied diverse scheduling models to select an appropriate participant client in the learning process at each round. In contrast, the authors in [42] prefer to choose high-data quality clients, ensuring system efficiency and prioritizing the clients who have suitable data rates rather than those with poor calculation and transmission capacities. So, it optimizes on-device data quality across clients while reducing delay, energy consumption, and packet size. Moreover, it provides a higher level of accuracy while improving convergence speed. Extremely dynamic scenarios were ignored in [8], where the average amount of resources and the required time for updating and uploading fluctuate dynamically. It assumes the scheduler has a pre-known local training time, which may only be realistic in some cases. It ignores client waiting time and undervalues the client’s latency in a global iteration. Moreover, transmission resource management and client data quality were neglected, and it could not decline the number of choices for clients with poor-quality data, leading to low global model accuracy and slow convergence. It only evaluates communication time, which accounts for a considerable amount of time for a training round.

- **Random methods**

In random selection, which is the conventional and basic form of CS, resource constraint issues [87,88] contain bandwidth allocation issues [5], limited computational resources issues [42], and the energy consumption of selected clients [26,42], which can lead to low accuracy and high convergence time and latency. In [26], the authors offered a novel strategy to choose fewer clients in earlier global iterations and more clients in later global iterations in the same period of training. This can increase model accuracy and reduce training loss when compared to choosing more clients at first. Because it overlooks the local data quality of clients and cannot decrease the number of selections for clients with low-quality data, the global model needs to be more accurate, and convergence needs to be faster. Neither CS nor resource management solutions were discussed in terms of how they affect the convergence and accuracy of global models. Likewise, [87] ignores the client data quality, so it is unable to decline the clients selected with low-level data quality and does not consider the client waiting time, leading to client latency. However, it considers client channel conditions and the importance of their local model updates. The authors studied diverse scheduling models to select an appropriate participant client in the learning process at each round. In contrast, the authors in [42] prefer to choose high-data quality clients, ensuring system efficiency and prioritizing the clients who have suitable data rates rather than those with poor calculation of transmission capacities. So, it optimizes on-device data quality across clients while reducing delay, energy consumption, and packet size. Moreover, it provides a higher level of accuracy while improving convergence speed.

- **MAB methods**

Multi-armed bandit-based method side effects are divided into four groups: dynamic wireless environment [93], client heterogeneity [7], data quality [52,91], and fairness [35,62,92]. In each sub-group, their main characteristic is the training latency [91,92,93]. To illustrate, authors in [93] proposed a CS algorithm based on the UCB policy and virtual queue technique (CS-UCB-Q). The method considers the availability of clients during FL training in the study because of the deep fade concern in wireless channels in both ideal and non-ideal strategies and unbalanced data in a volatile environment. However, the mentioned method and [1,7], cannot run asynchronously. In contrast, [91,92] can run asynchronously and provides a trade-off between training efficiency and fairness. A CS framework (AUCTION) as a model to obtain a root of fairness is suggested by [38], which employs a heuristic method to characterize the quality of each client and analyze the data quality challenges of each client in terms of the mislabeled and non-IID data. It is robust, adjustable, and scalable in diverse learning tasks and makes CS easy and flexible by automatically knowing procedures for variable client scales. Moreover, the research [38] develops a procedure network based on the encoder–decoder structure, which can be adjusted to dynamic modification clients and make sequential CS decisions to decrease RL searching space significantly. However, it did not consider the transmission expense of clients and computing latency to expand its CS functionality further. In another paper [35], a deadline-based aggregation model was offered to handle FL aggregation in a changing training environment, reaching faster convergence to fixed model accuracy. However, low-priority clients were denied training. Therefore, inequality selection does not guarantee data diversity on the global model aggregation. It ignores the local data quality of the clients, and it cannot decline the selections count for clients with low-quality data, resulting in low-level global model performance and slow convergence. The first research in mixing Lyapunov optimization and the C2MAB long-term constrained online scheduling issue is [1], which is a fairness-based CS while ensuring training efficiency and minimizing the average model exchange time when it is subject to a relatively flexible long-term fairness guarantee. It can handle unfair CS and large bias in data distribution, but it is unable to follow the theoretical analysis of the fairness for FL from the literature. It ignores the data quality factors, including mislabeling or non-IID, and it cannot find a way to trade-off between fairness and accuracy. It blindly considers fairness restrictions for each client while ignoring their contributions. Fairness quota metrics can severely impact training efficiency and should be assigned before training. Furthermore, it cannot run asynchronously. The authors of [62] introduce cumulative effective participation data (CEPD) as an optimization objective of volatile CS. They designed and implemented a CMAB model for learning efficient client participation and derived a finite constant upper bound on T-step regret based on UCBGS; however, they did not analyze the effect of policy fairness on training, nor the trade-off between fairness and overall training performance in a volatile FL. They also avoided focusing on selection adaptation when new clients are added. Overall, our findings indicate that MAB aims to minimize training latency. Considering an ideal and a non-ideal situation, it contains both local computation and data transmission times. The ideal scenario involves clients possessing IID datasets and always being available, whereas the non-ideal scenario involves clients being unavailable and the datasets being distributed non-IID. The primary purpose of the dynamic client sampling method is to improve the convergence rate. A non-convex training time minimization problem is developed by dynamic client sampling that gives an upper bound on convergence for arbitrary CS possibilities. Adopting such strategies can achieve the same target loss faster than the baseline. Using clustered sampling, different clients can be selected with different data allocations. An unbiased clustered sampling strategy for CS is offered that declines the weight variance of clients for the aggregating and provides unique client distribution. According to the authors, clustered sampling techniques were utilized for sample size and client similarity, so there is faster and better homogeneity with clustered sampling, especially for non-IID data.

Table 4 summarizes the advantages and disadvantages of the mentioned methods that were extracted through RQ2.

## 6. Limitations and Research Possibilities

To highlight motivations for future work, we first identify the limitations of current work and then discuss the critical potential points that should be considered for future work. This field of research is in its early stages, and there is limited research in literature. Hence, this work has the limitation of the number of reviewed publications. However, it should be considered that this is the first step to creating a comprehensive overview of this field. There are numerous unresolved concerns and issues surrounding CS in both the cross-silo and cross-device settings. This field of study presents numerous examination possibilities that need more in-depth analysis. In addition to developing high-performance CS algorithms for diverse application systems, existing work supposes the following issues as future open directions:

1. **Privacy and Communication:** In the FL process, the communication between clients and parameter servers usually occurs over an impaired wireless channel, which introduces some research queries about privacy issues and how the updates can be transferred to a secure channel.
2. **Trade-off between metric factors:** A considerable number of factors to improve model performance were used. However, different factors are not comparable. So, a need exists to balance factors for performance evaluation among various techniques for the same problem. For instance, selecting more clients in each training round boosts model performance and training efficiency but does not guarantee time efficiency, especially in a volatile environment. In the research that was reviewed in the paper, the rate of volatility in that space was unclear. This issue can be a potential research gap for future researchers.
3. **Asynchronous communication schemes:** Regarding analysis approaches, asynchronous communication schemes for local data updates remain an open issue demanding additional examination.
4. **Communication resource handling:** There is space to explore appropriate communication resource methods for allocating resources (same or different bandwidth, energy, and computational capacity) based on the network topology. This strategy can remarkably affect learning performance. This issue becomes essential when many client devices join the FL process. Remarkably, the training rate can be greatly reduced due to different client heterogeneity of computational capacities and data qualities. A favorable answer would be developing additional parts to encourage clients to use high-quality training data.
5. **Channel characteristics:** Analyzing the network requirements impacts the accuracy of federated model training. It is a future examination direction, particularly in wireless communication, when noise, path loss, shadowing, and fading impairments exist.
6. **Available datasets for clients:** The availability of client datasets is needed to obtain suitable training performance. Clients needed to use feature extraction for their local training. In this regard, one of the critical problems is the non-IID matter, potentially causing the local training to be highly divergent. Therefore, some solutions to cope with this matter need to be developed.

## 7. Conclusions

This paper provides a comprehensive SLR of FL in IoT devices and CS methods and their challenges. FL faces severe challenges, including expensive and inefficient communication, statistical heterogeneity, poor data quality, privacy concerns, and client heterogeneity. Based on the reviewed literature, CS is a suitable solution to these challenges. To better understand the importance of CS in FL, a categorization of CS methods, including clustering, random selection, greedy selection, and multi-armed bandit was presented. However, these methods contain some side effects, such as fairness, dynamic environment, and trustworthiness issues. Hence, finding a suitable CS method is still an open problem, and further exploration is needed. As a result, based on this work, it is possible to classify existing CS methods, understand their current status, and plan and move to develop more desirable and efficient approaches.

## Figures and Tables

**Table 1:** Main focuses and features of different surveys that exist in literature. (✕: Include criteria. ✓: Do not include criteria).

Criteria/	References												
	[6]	[18]	[19]	[24]	[27]	[32]	[36]	[38]	[41]	[53]	[54]	[55]	This Work
**Systematic Literature Review**	✕	✓	✕	✕	✕	✓	✕	✕	✕	✕	✓	✕	✓
**Focusing on the CS issue**	✓	✕	✕	✕	✕	✕	✓	✕	✕	✕	✕	✕	✓
**Compare the Pros and Cons of other papers**	✓	✕	✕	✕	✕	✓	✓	✕	✕	✕	✕	✕	✓
**Compare methods of CS**	✓	✕	✕	✕	✕	✕	✕	✕	✕	✕	✕	✕	✓
**Client heterogeneity issues discussion**	✓	✓	✓	✓	✕	✓	✓	✓	✓	✓	✕	✓	✓
**Data heterogeneity issues discussion**	✓	✓	✓	✕	✕	✓	✓	✓	✓	✓	✕	✕	✓
**Fairness issues discussion**	✓	✓	✕	✕	✕	✕	✕	✕	✕	✕	✕	✕	✓
**Dynamicity issues discussion**	✓	✕	✕	✕	✕	✕	✕	✕	✕	✕	✕	✕	✓
**Trustworthiness issues discussion**	✓	✕	✕	✓	✓	✕	✕	✕	✕	✕	✕	✕	✓
**Data Privacy issues discussion**	✕	✓	✓	✓	✓	✓	✕	✓	✓	✓	✓	✓	✓
**CS Categories**	✓	✕	✕	✕	✕	✕	✕	✕	✕	✕	✕	✕	✓
**Designing an architecture for CS**	✕	✓	✕	✕	✕	✕	✕	✕	✕	✕	✕	✕	✓

**Table 2:** A summary of the existing studies based on the defined RQs.

Research Question	Refs. No	Research Type
**RQ1.1**	[38,41,55]	Review
	[18,54,57]	SLR
	[8,28,33,34,39,58,59,60]	Experimental research
	[6]	Survey
**RQ1.2**	[18,61]	SLR
	[5,8,37,50,51,57,58,62,63,64,65,66,67,68,69,70,71,72,73,74,75,76,77,78,79,80,81,82,83]	Experimental research
	[36]	Survey
	[19,41]	Review
**RQ2.1**	[6,41,55]	Review
	[1,2,4,5,7,8,10,13,26,28,31,33,34,35,40,42,49,52,60,62,84,85,86,87,88,89,90,91,92,93]	Experimental research
	[6,36]	Survey
**RQ2.2**	[18]	SLR
	[1,2,5,8,13,28,33,34,35,40,49,62,84]	Experimental research
	[38]	Review

**Table 3:** CS Categories based on the probability of selection.

CS Methods	CS Challenges	Ref. No.	Main Characteristics	Applications	Strategy	Evaluation Metrics
Clustering	statistical heterogeneity	[28]	Non-IID data	Mobile/the IoT devices	CFL	Accuracy, F1 score, Micro-Acc, Micro-F1 Macro-Acc, Macro-F1
		[89]		Mobile phones/IoTs	Federated SEM	Ac, Convergence speed, Communication round
		[90]		Wireless edge	Federated MF	Accuracy, Convergence speed, Communication round
		[31]		Recommender systems	Iterative FCA	Accuracy
		[13]		Industrial IoT	Gradient-based Binary P-CS	Accuracy, Convergence speed
		[10]	Unbalanced Data	Recommendation systems	3-step modular solution	Iteration count
	Client Heterogeneity	[49]	Statistical heterogeneity communication cost	Mobile Phones	One-Shot FC, k-FED	Accuracy, Convergence speed
		[4]	Computation and communication cost issues	Mobile and IoT devices	FL with HC	Accuracy, Convergence speed, training round
		[2]		IoT systems- wireless devices	THF	Accuracy, Convergence speed
Greedy Selection	Client Heterogeneity	[40]	Bandwidth allocation issues	Mobile devices	Wireless FL network (WFLN)	Accuracy, Convergence speed
		[33]	Communication cost issues	Wireless communication	dynamic sampling	Accuracy
		[8]	Computational resources	MEC	Fed CS	Accuracy
		[85]	Convergence time communication computation constraint	IoT devices	Online Hybrid FL	Accuracy
Random Selection	statistical heterogeneity	[86]	Bandwidth allocation	Wireless FL system		Accuracy, Latency
		[42]	Energy consumption, delay, computation cost issues	Edge networks	A data-centric CS, DICE	Accuracy, Training round, Training time
	Client Heterogeneity	[26]	Energy consumption, latency issues	IoT	ELASTIC	Number of selected clients and energy consumption
		[5]	Bandwidth allocation issues	IoT networks	FL in fog-aided IoT ALTD	Accuracy, Convergence speed
		[87]	Resource allocation- Convergence issues	MUEs	Scheduling and resource allocation	Accuracy, Convergence speed
		[88]	Model training efficiency, resource constraints		Stochastic integer CS	Accuracy
Multi-armed bandit-based Selection	Client Heterogeneity	[93]	Training latency, dynamic wireless environment	Wireless networks	A CS based on UCB and queue	Accuracy, Convergence speed
		[7]	Training latency		Online scheduling scheme	Accuracy, Training latency
		[84]	Convergence issues and Volatility	IoT devices	CE Participation Data	Accuracy, Convergence speed
	statistical heterogeneity	[52]	Data Quality (Mislabeled and non-IID)	Wireless networks	AUCTION	Accuracy, Scalability
		[91]	Training performance communication time	Mobile devices	Context-aware Online CS	Accuracy, Convergence speed
	Fairness	[35]	Training efficiency	IoT	(Exp3)-based CS	Accuracy, the communication rounds
		[34]	Convergence speed-the training latency	IoT	CEB3	
		[1]			Fairness-guaranteed, RBCS-F	Accuracy, Training time
		[62]			UCB-GS	Communication and computational cost, Execution time
		[92]			FLACOS	Accuracy, Convergence speed, Training time

**Table 4:** The advantages and disadvantages of each CS method.

CS Strategies	Advantages	Disadvantages
Random	Clients’ similar selection chance. Controllable sampling data.	Neglecting the client data quality. Latency. Unable to reduce the number of clients selected with low-quality data. Increased energy consumption. Inefficient aggregation model.
Greedy	Improved data quality. Each client’s effective participation is known in advance. Optimal time running.	Underestimated client latency The risk of finding the optimal clients. Difficulties in obtaining accurate resource information for all clients before the FL process.
Clustering	Reduced variance of the local and global models. Reduced communication costs and communication rounds. Reduced network congestion. Reduced device failure.	Hard to tune clusters. Difficulties in obtaining accurate resource information for all clients before the FL process. Difficulties in the scalability of the infrastructure. Extra overheads.
MAB	Able to find clients with rich resources. Fairness in selecting clients. Alleviates bandwidth, time, and computation limitations. Remembers models and stops repeated models. Increased the convergence speed. Uncertainties for decision making. Balanced CS.	Computational complexity.

## Data Availability

Not applicable.

## Abbreviations

**AI**	Artificial intelligence	**MAB**	Multi-Armed Bandit
**AUC**	Area Under curve	**Macro-Acc**	Macro-Accuracy
**AUROC**	Area Under the ROC curve	**MEC**	Mobile edge computing
**CEPD**	Cumulative effective participation data	**Micro-Acc**	Micro-Accuracy
**CFL**	Clustered FL	**ML**	Machine learning
**CMAB**	Combinatorial multiarmed bandit	**MUEs**	Mobile user equipment
**CS**	Client selection	**non-IID**	Non-independent and -identical data
**FedAvg**	Federated averaging	**ROC**	Receiver Operating Characteristic
**FedCS**	Federated client selection	**RQs**	Research questions
**FL**	Federated Learning	**RS**	Recommendation systems
**FN**	False Negatives	**SGD**	Stochastic gradient descent
**FP**	False Positive	**THF**	3-way hierarchical
**GBP- CS**	Gradient-based Binary Permutation Algorithm	**TN**	True Negative
**IIoT**	Industrial Internet of Things	**TP**	True Positive
**IoT**	Internet of Things	**MAB**	Multi-Armed Bandit
**IoV**	Internet of Vehicles	**WFLN**	Wireless FL network

## Appendix A

The assessment of FL can be categorized into two distinct dimensions: model performance and system performance. The evaluation of model performance entails quantification through metrics convergence and accuracy. Accuracy is also allied with measures such as Recall, Precision, F1-Score, Micro-Acc, Micro-F1, Macro-Acc, and Macro-F1. These metrics serve as valuable methodologies to gauge the effectiveness of individual clients’ contributions to the overall FL system.

The evaluation of model convergence is achievable through multiple facets, encompassing criteria such as training loss, the count of communication rounds, the number of local training epochs, and the establishment of formal convergence boundaries. Conversely, the assessment of system performance metrics directs its attention to parameters such as communication efficiency, computational efficiency, system heterogeneity, system scalability, and the capability to withstand faults [93,94]. These metrics are elucidated in greater depth in the subsequent paragraph.

➢ Model performance metrics
  - **Accuracy**

Accuracy pertains to the proportion of correctly classified instances within the test set. Throughout the annals of machine learning research, accuracy has wielded considerable influence as a performance metric [28].

(A1) $$Accuracy=\frac{TP+TN}{FP+FN+TP+TN}$$

TP signifies the count of instances that have been accurately forecasted as positive by the model.

TN quantifies the instances that have been correctly predicted as negative.

FP delineates instances that have been erroneously categorized as positive. FN accounts for instances that have been inaccurately classified as negative by the model.

- **Precision**

Precision assesses the veracity of positive predictions generated by the model. It involves the computation of the proportion of true positive predictions relative to the total instances that have been predicted as positive (sum of true positives and false positives). In essence, precision provides insight into the fraction of positive predictions that have been accurately determined. Elevated precision signifies that the model demonstrates a reduced frequency of false positive predictions [95].

(A2) $$Precision=\frac{TP}{FP+TP}$$

- **Recall**

Referred to as sensitivity or true positive rate, this metric gauge the model’s competence in apprehending all factual positive instances. It quantifies the correlation between true positive predictions and the entirety of actual positive instances (sum of true positives and false negatives). In simpler terms, recall provides insight into the percentage of positive instances that have been accurately anticipated as positive. A heightened recall signifies that the model adeptly identifies a significant portion of positive instances [95].

(A3) $$Recall=\frac{TP+TN}{FP+TP}$$

- **F1-Score**

The F1-score represents the harmonic mean achieved by integrating precision and recall, amalgamating these two metrics into a solitary value to offer an equilibrium-based gauge of the model’s performance [28].

(A4) $$F1-score=2*\frac{Precision\times Recall}{Precision+Recall}$$

- **Micro-Acc**

Micro-Acc is an evaluation measure deployed in the context of multi-class classification endeavors. It serves to compute comprehensive accuracy by aggregating the accurate predictions across all classes. This approach conceptualizes the problem akin to a binary classification scenario, where the affirmative class signifies correct predictions, while the negatory class denotes incorrect ones. This metric accords equal significance to each individual instance [28].

(A5) $$Micro-Acc=\frac{TP1+TP2+\ldots TPn}{TP1+TP2+\ldots TPn++FP1+FP2+\ldots +FPn}$$

TPi denotes the numerical representation of true positive instances exclusively associated with class I.

FPi signifies the cumulative enumeration of false positive instances linked to class I.

n denotes the entirety of classes that are presently being considered [28].

- **Macro-Acc**

Macro-Acc constitutes an additional assessment metric that finds application in the realm of multi-class classification endeavors. In contrast to micro-accuracy, which accords equal significance to each individual instance, macro-accuracy ascertains the mean accuracy for each distinct class. Subsequently, it computes the average of these accuracies pertaining to individual classes, thereby deriving a holistic evaluation of the model’s performance.

(A6) $$Macro-Acc=\frac{Acc1+Acc2+\ldots +Accn}{n}$$

Acci represents the accuracy pertaining to class I.

n signifies the total count of classes encompassed [28].

- **Micro-F1**

Micro-F1 is determined through an inclusive process encompassing all instances, along with their respective true positive, false positive, and false negative tallies across various classes. These data are subsequently employed to compute precision and recall. The ultimate Micro-F1 score materializes as the harmonic mean of micro-precision and micro-recall. Micro-F1 effectively addresses the challenge of class imbalance according to equal weight in all instances. This metric finds pertinence in scenarios wherein an overarching assessment of model performance across diverse classes is sought, with no bias towards larger classes.

(A7) $$Micro-F1=\frac{2*(Micro-Precision*Micro-Recall)}{Micro-Precision+Micro-Recall}$$

where

$$Micro-Precision=\frac{TP\_total}{TP\_total+FP\_total}$$

$$Micro-Recall=\frac{TP\_total}{TP\_total+FN\_total}$$

TP_total: The cumulative count of true positives across all classes.

FP_total: The cumulative count of false positives across all classes.

FN_total: The cumulative count of false negatives across all classes [28].

- **Macro-F1**

Macro-F1 is ascertained through an initial process involving the independent computation of F1-scores for individual classes, followed by the aggregation of these class-specific F1-scores to derive an average. Every class’s F1-score carries identical weightage in this computation, irrespective of the class’s magnitude. This metric equipped an equitable assessment of the model’s efficacy spanning all classes. It guarantees uniform consideration to each class, an attribute particularly advantageous when evaluating the model’s adeptness in dealing with smaller classes [28].

(A8) $$Macro-F1=\frac{\left(F1\_class1+F1\_class2+\ldots +F1\_classn\right)}{n}$$

where

$$F1\_classi=\frac{2*(Precision{\_class}_{i}*Recall\_classi)}{\left(Precision\_{class}_{i}+Recall\_classi\right)}$$

Precision_class_i_ corresponds to the precision value attributed to class I.

Recall_class_i_ pertains to the recall value pertaining to class I.

n denotes the total count of classes in consideration.

- **AUC**

One metric to consolidate the ROC curve into a single metric involves the calculation of AUROC, often denoted as AUC. This metric carries a well-established statistical interpretation, specifically defined as the probability that a randomly chosen instance from a particular class demonstrates a lower estimated likelihood of belonging to the opposing class in comparison to a randomly chosen instance from the opposing class [95].

(A9) $$AUC=\sum_{i=1}^{\infty }\frac{\left(TPRate[i]+TPRate[i-1])*(FPRate[i]-FPRate[i-1]\right)}{2}$$

True Positive Rate[i] designates the true positive rate, also known as sensitivity, observed at the i-th threshold.

False Positive Rate[i] signifies the false positive rate, denoted as 1 minus specificity, as assessed at the i-th threshold.

- **Convergence speed**

By having a faster convergence speed on clients, local models adapt quickly to their respective datasets and contribute effectively to global model improvement. During the assessment of model performance, it encompasses the subsequent metrics:

▪ **Training time/training duration**

It denotes the actual time taken by each distinct client to conduct localized training utilizing its local dataset. In every training round, each client engages in the training of a model on its local data, thereby strengthening the model underlying parameters. This metric encompasses the cumulative time needed for executing multiple localized epochs on the client dataset. This metric serves as an assessment of the computational exertion entailed in the process of enhancing the model on each client [92].

▪ **Training loss**

It signifies the measurement of the discrepancy between the model projected outcomes and the factual ground truth during the localized training phase executed on each client. This metric of loss functions as an indicator of the model’s congruence with the intended target results and assumes a guiding role in the optimization procedure aimed at reducing the variance between predictions and factual values.

▪ **Training round (number of local training epochs)**

Its emphasis lies in the procedure of revising model parameters on a particular dataset through repeated iterations. This represents a foundational concept within the realm of machine learning, extending its applicability to both conventional training methodologies and the domain of federated training.

➢ **System performance metrics**
  - **Convergence speed**

The concept of convergence speed observed at the server level indicates the rapidity with which the amalgamated global model, derived from client updates, progresses toward optimal performance. This aspect is related to the speed at which the aggregation procedure, completed at the server level, combines the diverse client model updates. A rapid convergence speed on the server emphasizes the proficient integration of diverse client contributions. This issue facilitates accelerated attainment of convergence for the global model. It contains the following metrics:

▪ **Execution Time**

Execution time in FL encapsulates the complete duration required for an entire iteration round. An iteration round involves multiple phases, including distributing the global model to the client, conducting local training on each client, aggregating model updates, and generating a new global model. Execution time takes into account not only the training time on each client but also the time needed for communication, aggregation, and synchronization between the central server and clients [62,96].

▪ **Iteration count**

It pertains to the frequency of iterations through which the training process is iteratively executed across the clients. FL encompasses the cooperative training of a model across a multitude of devices while upholding the data’s localization on these devices as opposed to its centralization. The iteration count in federated learning encompasses the entire cycle of communication, local training, and model aggregation across all client devices. It refers to the number of times this complete cycle is repeated. Each iteration consists of distributing the global model to clients, clients performing local training, aggregating model updates, and generating a new global model. The iteration count represents the number of times this process is repeated until convergence [10].

- **Communication efficiency**

Assessing communication efficiency involves scrutinizing metrics such as the count of communication rounds, the tally of parameters, and the sizes of transmitted messages. Communication rounds facilitate data exchange between clients and servers in a training network. This measure provides a quantitative assessment of models trained with data from different clients [35].

- **Computational efficiency**

The evaluation of computational efficiency encompasses the examination of metrics including the duration of training. This assessment pertains to the computational resources essential for model training, encompassing aspects such as CPU and GPU utilization, memory consumption, and other hardware-related considerations to handle latency arising from non-IID data [62].

- **System scalability**

Indicates the capacity of a model to effectively manage growing quantities of data, workload, or users while maintaining its performance at a satisfactory level. The assessment of system scalability involves the analysis of its efficacy across an extensive array of clients, encompassing criteria such as performance outcomes, overall time taken, and aggregate memory usage [52].
